# Early mortality risk prediction in severe fever with thrombocytopenia syndrome using an interpretable machine learning model based on routine clinical parameters

**DOI:** 10.3389/fpubh.2026.1776344

**Published:** 2026-03-09

**Authors:** Qian Dai, Ji Guo, Liangfei Xu, Qiong Lu, He Chen, Yuanyuan Hu, Ying Wang, Tong Tong

**Affiliations:** 1 Department of Clinical Laboratory, The First Affiliated Hospital of Anhui Medical University, Hefei, China; 2 Department of Blood Transfusion, Chaohu Hospital Affiliated to Anhui Medical University, Hefei, China; 3 Department of Laboratory Medicine, The First Affiliated Hospital of USTC, Division of Life Sciences and Medicine, University of Science and Technology of China, Hefei, China

**Keywords:** Dabie bandavirus, explainable artificial intelligence, machine learning, prognostic prediction, SFTS

## Abstract

**Background:**

Severe Fever with Thrombocytopenia Syndrome (SFTS) is characterized by high mortality and rapid progression, necessitating accurate early prognosis to optimize supportive care. However, current predictive tools often lack interpretability, require sophisticated tests unavailable in resource-limited areas, or suffer from poor generalizability. This study aimed to develop an interpretable, parsimonious, and deployable machine learning model for early mortality prediction in SFTS.

**Methods:**

We analyzed data from 834 SFTS patients across three medical centers in Anhui, China. A LightGBM model was developed using a derivation cohort (*n* = 571) and validated on internal (*n* = 143) and two independent external cohorts (*n* = 80 and *n* = 183). Model interpretability was enhanced using SHapley Additive exPlanations (SHAP), and a web-based calculator was deployed for clinical use.

**Results:**

The LightGBM model identified six routine clinical parameters—Age, Lactate Dehydrogenase (LDH), Activated Partial Thromboplastin Time (APTT), Uric Acid (UA), Creatinine (CRE), and Body Temperature—as the most influential predictors. Integrating these features, the model achieved robust discrimination with an Area Under the Curve (AUC) of 0.960 in the training set and 0.938 in the internal validation set. Crucially, it maintained strong performance in two independent external validation cohorts (AUC 0.871 and 0.877). SHAP analysis revealed that Age and LDH were the strongest risk factors, while Temperature exhibited a non-linear relationship with mortality risk.

**Conclusion:**

We developed and validated a high-performance, interpretable ML model for SFTS prognosis relying on only six readily available parameters. By deploying this parsimonious model as an online calculator, we provide a practical decision-support tool to facilitate early risk stratification and timely intervention, particularly in resource-limited settings.

## Introduction

Severe Fever with Thrombocytopenia Syndrome (SFTS) is an emerging tick-borne viral disease caused by the novel bunyavirus (SFTSV) (1, 2). First identified in China in 2009, the disease has since been reported in several East Asian countries, including Japan, South Korea, Thailand, and Pakistan (3). The recent detection of its primary vector, Haemaphysalis longicornis, in the United States, Russia, Australia, and Western Pacific regions indicates a potential global spread, raising SFTS to a significant public health concern (4–7). The clinical course is often severe, with critically ill patients frequently progressing to multiple organ failure within 2 weeks of onset, and case fatality rates ranging from 5.2 to over 30% (8). To date, no approved vaccines or specific antiviral treatments are available (9). Clinical management relies primarily on early and comprehensive organ function support, the efficacy of which is highly time-dependent. Therefore, accurate prognosis prediction at an early clinical stage is crucial.

Ticks predominantly inhabit mountainous and forested areas, leading to a concentration of SFTS cases in rural and remote regions (10). This epidemiological pattern presents two major challenges for prognosis management: (1) Primary healthcare facilities in these areas often lack simple yet effective tools to identify high-risk patients requiring urgent referral. This, compounded by limited resources, frequently results in delayed critical care during the crucial treatment window, adversely affecting outcomes. (2) Even in well-equipped tertiary hospitals, clinicians need to accurately identify patients at risk of clinical deterioration soon after admission. Early identification enables timely initiation of intensive treatments—which may include specific antiviral agents, immunomodulatory therapy, and continuous renal replacement therapy (CRRT)—while also facilitating early communication with families and rational allocation of monitoring resources.

Traditional scoring systems such as SOFA and APACHE II demonstrate limited predictive performance for SFTS, as their linear assumptions fail to capture the disease's complex progression (11). Machine learning (ML) presents a promising alternative, showing superior ability to model non-linear relationships and improve prognostic accuracy (12, 13), with several studies confirming its advantage over conventional methods in predicting SFTS outcomes (14–17).

However, existing ML models exhibit significant limitations in addressing the aforementioned clinical challenges. A primary concern is the “black-box” nature of many models, where opaque decision-making hinders clinical interpretability and trust, limiting their use in critical decisions (18). Furthermore, many high-performing models rely on complex predictors or laboratory parameters, such as nucleic acid testing, that are not readily available in primary care settings (14), reducing their feasibility at the grassroots level. Additionally, some studies are constrained by insufficient sample sizes (15), undermining model generalizability and reliability.

To address these limitations, we developed and validated an interpretable ML-based prognostic model using multi-center data. The model integrates baseline vital signs, comorbidities, and laboratory parameters to minimize retrospective bias (19). Employing advanced feature selection on a cohort larger than those in previous studies, (14, 16) we identified a concise set of accessible predictors. SHapley Additive exPlanations (SHAP) were implemented to provide global and local interpretability (18). The model's robustness and generalizability were confirmed through rigorous internal and external validation. Finally, we deployed an accessible online calculator that operates without requiring nucleic acid test results, offering a rapid, reliable, and low-cost tool for immediate clinical application in SFTS prognosis.

This model is designed to achieve two primary clinical objectives: first, to provide primary care physicians with an objective, quantifiable tool for referral decision-making, enabling rapid risk assessment and timely transfer of high-risk patients to prevent critical treatment delays; second, to offer early warning for clinicians in receiving hospitals, facilitating the accurate identification upon admission of patients requiring intensive monitoring and active intervention, thereby providing a prospective reference for treatment optimization. Through this research, we aim to establish an early warning system capable of assessing mortality risk in SFTS patients at an early stage, ultimately promoting tiered diagnosis and treatment, enabling precise management, and improving patient outcomes.

## Method

### Study population and data sources

This retrospective multicenter cohort study aimed to develop and validate a prognostic model for patients with Severe Fever with Thrombocytopenia Syndrome (SFTS). The derivation cohort included 571 patients from the First Affiliated Hospital of Anhui Medical University (January 2022 to January 2025). Two independent external validation cohorts comprised 80 patients from the First Affiliated Hospital of University of Science and Technology of China (December 2023 to August 2025) and 183 patients from Chaohu Hospital of Anhui Medical University. The primary endpoint was all-cause mortality. Survival status at discharge was obtained from electronic medical records, supplemented by telephone follow-up within 30 days post-discharge for cases with uncertain outcomes. The study was approved by the Ethics Review Committee of Chaohu Hospital of Anhui Medical University (Ethics No. KYXM-202509-003), the Ethics Review Committee of the First Affiliated Hospital of Anhui Medical University (Ethics No. PJ-2025-07-44) and the Ethics Review Committee of The First Affiliated Hospital of USTC (Ethics No. 2025-RE-363). Due to the retrospective design, patient informed consent was waived, and all personal identifiers were removed.

### Inclusion and exclusion criteria

Inclusion criteria were: 1) Diagnosis of SFTS per Chinese guidelines (2023), defined by acute fever (>38 °C) with thrombocytopenia (< 1001 × 10^9^/L) and/or laboratory confirmation via qRT-PCR, viral isolation, or seroconversion; 2) Hospitalized adults (≥18 years); 3) Complete baseline and outcome data. Exclusion criteria were: 1) >15% missing data; 2) Indeterminate outcomes; 3) Confirmed co-infection with other pathogens (e.g., COVID-19, Hantavirus); 4) Pregnancy or major comorbidities significantly affecting prognosis (e.g., active autoimmune disease, malignancy).

### Data collection and preprocessing

Demographic information, vital signs, and laboratory results at the time of admission were gathered from the electronic medical record system. The extent of missing data for each variable is detailed in Supplementary Table S1. Generally, the dataset exhibited a high degree of completeness; most variables had a missing rate of less than 2%, with the highest missing rate being 5.25% (Glucose). Given this low percentage of missingness, for categorical variables with missing data, the mode was utilized for imputation, whereas the median method was employed for continuous variables that had missing values (20). To minimize the impact of different measurement scales on the model, all continuous variables were standardized by Z-score. To avoid the impact of missing data on model construction, features with more than 15% missing values were excluded.

To avoid omitting important variables, a total of 50 variables from multiple dimensions were collected. These 50 variables cover: 1) patient demographic characteristics (age, sex); 2) patients' baseline vital signs at admission: systolic blood pressure (SBP), diastolic blood pressure (DBP), body temperature, Beats Per Minute (BMP); 3) underlying diseases: hypertension, coronary heart disease (CHD), diabetes; 4) laboratory indicators at first diagnosis: WBC, ANC (Absolute Neutrophil Count), ALC (Absolute Lymphocyte Count), AMC (Absolute Monocyte Count), RBC, HGB, HCT, MCV, MCH, MCHC, PLT, CRP, TP, ALB, GLO (Globulin), A/G (Albumin to Globulin ratio), TBIL, ALT, AST, ALP, GGT (Gamma-Glutamyl Transferase), UREA, CRE, UA (Uric Acid), GLU, LDH, K, Na, CL, HCO3, LPS, AMY, PT, PT%, PT-INR, APTT, FIB, TT, DD, PCT, AST/ALT, UCR. Besides, the patient's outcome (survived or died) was used as the dependent variable.

### Feature selection

Least Absolute Shrinkage and Selection Operator (LASSO) regression with L1 regularization was applied to the training set for feature selection, promoting model sparsity and managing multicollinearity (21). The final predictor set was determined by intersecting features with non-zero coefficients under the λ.1se criterion and those significant in univariate analysis (Table 1).

**Table 1:** Baseline characteristics of the derivation cohort, stratified by survival outcome.

**Feature**	**Survived (*n* = 369)**	**Died (*n* = 142)**	***P* value**
Age (year), (mean ± sd)	67.00 (58.00–74.00)	73.00 (67.25–80.00)	< 0.001
**Sex**, ***n*** **(%)**			0.861
Female	227 (52.9%)	77 (54.2%)	
Man	202 (47.1%)	65 (45.8%)	
**Hypertension**, ***n*** **(%)**			0.257
No	336 (78.3%)	104 (73.2%)	
Yes	93 (21.7%)	38 (26.8%)	
**CHD**, ***n*** **(%)**			0.373
No	425 (99.1%)	139 (97.9%)	
Yes	4 (0.9%)	3 (2.1%)	
**Diabetes**, ***n*** **(%)**			0.621
No	363 (84.6%)	117 (82.4%)	
Yes	66 (15.4%)	25 (17.6%)	
Temperature ( °C), median (IQR)	38.69 (38.00–39.00)	39.00 (38.50–39.00)	0.002
BPM, median (IQR)	82.00 (74.00–89.00)	83.56 (75.00–95.00)	0.106
SBP, mean ± sd	115.00 (103.00–126.00)	119.00 (104.00–134.00)	0.051
DBP, mean ± sd	72.00 (65.00–79.00)	72.50 (62.00–82.00)	0.784
WBC (10^9^/L), median (IQR)	2.41 (1.78–4.48)	2.63 (1.78–4.56)	0.744
ANC (10^9^/L), median (IQR)	1.53 (0.98–3.13)	1.74 (1.18–3.34)	0.226
ALC (10^9^/L), median (IQR)	0.63 (0.42–0.97)	0.56 (0.37–0.91)	0.092
AMC (10^9^/L), median (IQR)	0.15 (0.08–0.28)	0.11 (0.07–0.23)	0.024
RBC (10^12^/L), median (IQR)	4.29 (3.92–4.65)	4.22 (3.76–4.78)	0.486
HGB (g/L), mean ± sd	130.00 (119.00–140.00)	127.00 (113.00–145.75)	0.452
HCT (%), median (IQR)	37.40 (32.10–40.90)	37.45 (31.95–42.30)	0.584
MCV (fl), median (IQR)	90.20 (87.40–93.30)	89.65 (86.88–92.88)	0.333
MCH (pg), median (IQR)	30.50 (29.40–31.50)	30.30 (29.20–31.30)	0.247
MCHC (g/L), mean ± sd	337.00 (329.00–344.00)	338.00 (328.25–346.00)	0.633
PLT (10^9^/L), median (IQR)	62.00 (44.00–84.00)	45.00 (33.25–64.75)	< 0.001
CRP, median (IQR)	3.29 (1.26–9.16)	7.47 (2.92–15.44)	< 0.001
TP (g/L), mean ± sd	63.90 (59.30–69.10)	62.20 (57.67–67.00)	0.014
ALB (g/L), median (IQR)	36.20 (32.60–40.00)	33.85 (30.27–37.50)	< 0.001
GLO (g/L), median (IQR)	27.90 (24.30–31.30)	28.55 (25.35–31.18)	0.265
A/G, median (IQR)	1.32 (1.12–1.55)	1.20 (1.01–1.41)	< 0.001
TBIL (μmol/L), median (IQR)	9.50 (7.51–12.40)	9.95 (7.83–13.10)	0.273
ALT (U/L), median (IQR)	49.00 (29.00–86.80)	67.00 (39.00–138.00)	< 0.001
AST (U/L), median (IQR)	98.00 (50.60–185.00)	186.00 (97.25–484.75)	< 0.001
ALP (U/L), median (IQR)	72.00 (56.00–77.00)	76.52 (56.47–81.00)	0.196
GGT (U/L), median (IQR)	26.00 (17.70–50.00)	30.00 (17.00–60.50)	0.243
UREA (mmol/L), median (IQR)	6.10 (4.59–7.85)	8.38 (5.92–11.29)	< 0.001
CRE (μmol/L), median (IQR)	73.80 (59.30–92.00)	88.60 (68.55–118.25)	< 0.001
UA (μmol/L), median (IQR)	266.00 (203.00–336.00)	312.00 (222.50–436.25)	< 0.001
GLU (mmol/L), median (IQR)	6.85 (5.74–8.27)	7.82 (6.31–9.88)	0.001
LDH (U/L), median (IQR)	520.00 (305.00–791.44)	791.44 (492.67–1,210.75)	< 0.001
K (mmol/L), mean ± sd	3.67 (3.38–3.96)	3.92 (3.50–4.30)	< 0.001
Na (mmol/L), mean ± sd	133.90 (131.50–136.70)	133.25 (130.75–136.07)	0.174
CL (mmol/L), median (IQR)	100.00 (97.00–103.40)	100.00 (96.03–103.78)	0.990
HCO3 (mmol/L), median (IQR)	22.80 (21.00–25.00)	21.25 (18.20–23.85)	< 0.001
LPS (U/L), median (IQR)	331.00 (125.00–553.00)	397.50 (258.25–693.50)	0.002
AMY (U/L), median (IQR)	101.00 (71.00–142.00)	122.00 (88.50–150.25)	0.002
PT (s), mean ± sd	12.60 (11.60–13.50)	12.85 (11.38–13.60)	0.481
PT%, mean ± sd	99.37 (90.00–110.20)	97.95 (89.00–103.22)	0.014
PT-INR, mean ± sd	0.99 (0.93–1.07)	1.00 (0.97–1.07)	0.018
APTT (s), median (IQR)	41.60 (36.50–48.70)	47.70 (41.62–57.45)	< 0.001
FIB (g/L), median (IQR)	2.65 (2.29–2.97)	2.48 (2.15–2.77)	0.002
TT (s), median (IQR)	21.00 (19.00–24.50)	24.60 (21.11–31.50)	< 0.001
DD (μg/ml), median (IQR)	2.04 (1.09–3.93)	4.73 (2.48–8.67)	< 0.001
PCT (ng/ml), median (IQR)	0.20 (0.10–1.86)	0.58 (0.24–1.86)	< 0.001
AST/ALT, median (IQR)	2.08 (1.60–2.68)	2.80 (2.15–3.67)	< 0.001
UCR, mean ± sd	19.89 (16.91–24.23)	22.24 (17.35–27.63)	0.012

IQR, interquartile range; sd, standard deviation; CHD, coronary heart disease; BPM, beats per minute; SBP, systolic blood pressure; DBP, diastolic blood pressure; ANC, absolute neutrophil count; ALC, absolute lymphocyte count; AMC, absolute monocyte count; A/G, albumin to globulin ratio; UCR, (UREA to CRE) ^*^ 250.

### Machine learning models

Five algorithms were trained and compared: logistic regression (LR), support vector machine (SVM), random forest (RF), eXtreme gradient boosting (XGBoost), and light gradient boosting machine (LightGBM). The dataset was split into training (70%) and internal testing (30%) sets. Model hyperparameters were optimized via 5-fold cross-validation on the training set. The final model was selected based on comprehensive performance evaluation across an external 5-fold cross-validation.

### Model evaluation and interpretation

Model performance was assessed using the area under the receiver operating characteristic curve (ROC-AUC) and precision-recall curve (PR-AUC), sensitivity, specificity, Brier score, and decision curve analysis. SHapley Additive exPlanations (SHAP) were used to provide global and local interpretability. Given the moderate class imbalance in our derivation cohort (24.9% mortality), we employed the Synthetic Minority Over-sampling Technique (SMOTE) to balance the class distribution of the training data. This technique addresses imbalance by creating synthetic instances of the minority class (mortality) rather than by simple duplication, thus providing a more diverse and balanced dataset for training. Crucially, in our cross-validation framework, SMOTE was applied only to the training set within each fold, while the corresponding validation set was left in its original, imbalanced state. This standard practice ensures that the model's performance is evaluated on a realistic, non-synthetic data distribution, preventing data leakage and providing a true assessment of its generalization capability. This balanced training approach was applied to all five machine learning models to ensure a fair comparison. The final model was deployed as an interactive web calculator using the Streamlit framework.

### Statistical analysis

Analyses were performed using SPSS (v27.0), R (v4.2.2), and Shiny (v0.5.1). Categorical variables are presented as percentages and compared using Chi-square or Fisher's exact test. Continuous variables are expressed as mean ± standard deviation or median (interquartile range) and compared using Student's *t*-test or Mann–Whitney *U*- test, as appropriate. A two-sided *p*-value < 0.05 was considered statistically significant.

## Result

### Patient clinical characteristics

**Figure 1:**
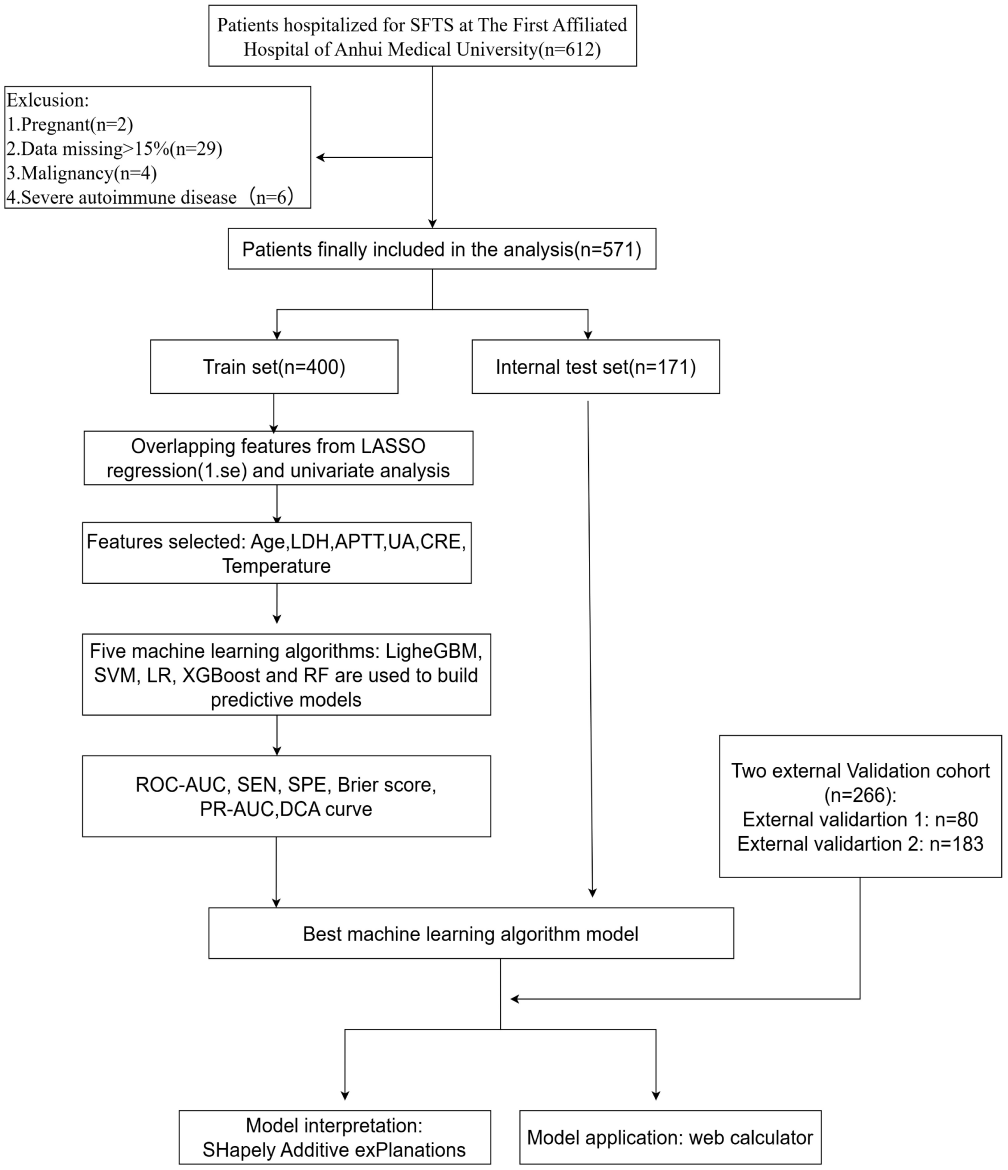
Flow chart of the study design.

This retrospective study enrolled a derivation cohort of 571 SFTS patients from the First Affiliated Hospital of Anhui Medical University, among whom 142 (24.9%) died. The cohort was randomly divided into a training set (70%) and an internal validation set (30%). Two external validation cohorts were also included: 80 patients from the First Affiliated Hospital of University of Science and Technology of China and 183 patients from Chaohu Hospital of Anhui Medical University. The patient selection process is summarized in Figure 1.

As presented in Table 1, patients who died were significantly older and demonstrated more pronounced laboratory abnormalities, including lower platelet counts and albumin levels, along with elevated inflammatory markers (CRP, PCT), organ injury indicators (LDH, AST, CRE, UREA), and coagulation parameters (APTT, D-dimer; all *p* < 0.001). Demographic and clinical characteristics of the external validation cohorts are provided in Supplementary Tables S2, S3.

### Selection of predictors

**Figure 2:**
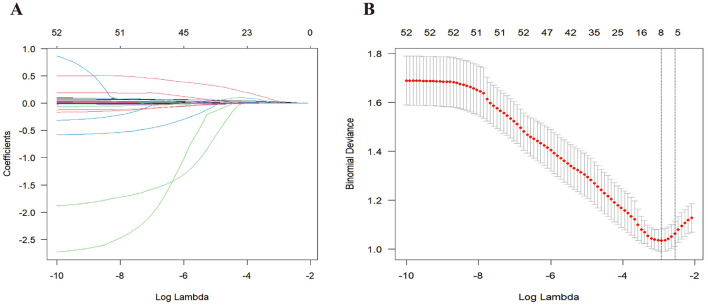
Feature selection using LASSO regression. **(A)** The bar plot displays the coefficients of features retained by the LASSO model; **(B)** The features are ranked according to the absolute values of their coefficients from top to bottom.

We applied LASSO regression to 50 candidate variables to identify key prognostic predictors, using coefficient shrinkage to mitigate overfitting and multicollinearity in train data set. Figure 2A illustrates the coefficient paths as log (λ) increased from −10 to −2, showing a progressive reduction in the number of retained features. Figure 2B showed through 5-fold cross-validation guided by the one-standard-error rule, six predictors were selected: age, LDH, APTT, UA, CRE, and Body temperature (Table 2). All six features had previously shown significant differences in univariate analysis (Table 1), supporting their relevance for prognostic modeling.

**Table 2:** Coefficients of the six features selected by the Lassso regression.

**Features**	**Coefficient**
Age	0.009396675
LDH	0.005972533
APTT	0.0021809
UA	0.000607394
CRE	0.000260473
Teprature	0.000117962

### Predictive performance of machine learning models

We developed and evaluated five machine learning models-LightGBM, SVM, RF, LR, and XGBoost-using six selected features. Model performance was assessed in terms of discrimination, precision-recall characteristics, and calibration on both training and internal test sets.

**Figure 3:**
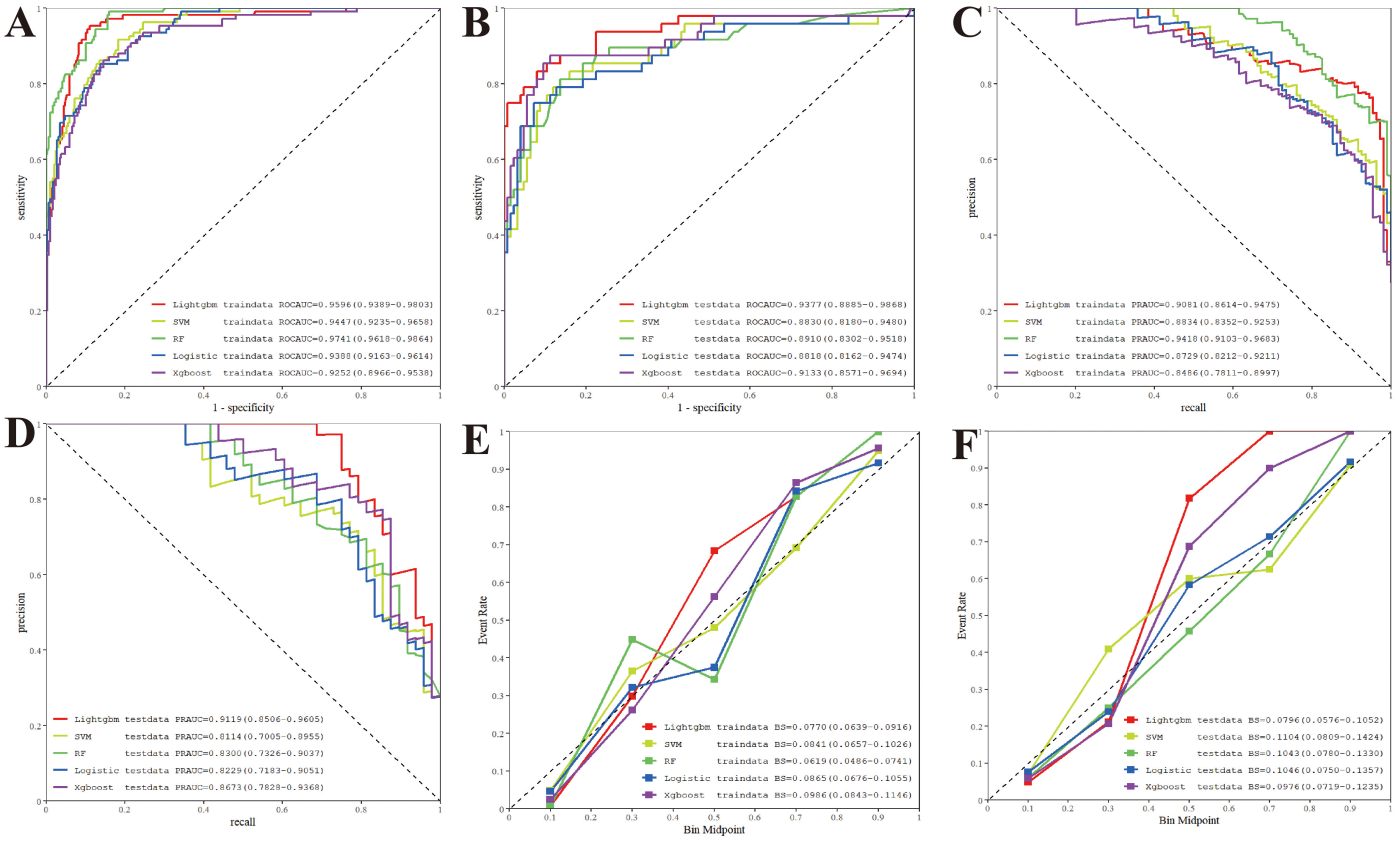
Performance metrics of different machine learning models on the internal validation set. **(A)** ROC curves of five models for train cohort; **(B)** ROC curves of five models for internal validation cohort; **(C)** PR analysis of five models for train cohort; **(D)** PR analysis of five models for internal validation cohort; **(E)** Brier score of five models for train cohort; **(F)** Brier score of five models for internal validation cohort.

On the training set, all models exhibited strong discriminative ability. RF achieved the highest ROC-AUC of 0.9741 (95%CI: 0.9618–0.9864), followed closely by LightGBM (0.9596), SVM (0.9447), LR (0.9388), and XGBoost (0.9252) (Figure 3A). In the internal validation set, LightGBM demonstrated the best generalization, attaining a ROC-AUC of 0.9377 (95% CI: 0.8885–0.9868), outperforming RF (0.8910), XGBoost (0.9133), SVM (0.8830), and LR (0.8818; Figure 3B).

Precision-recall (PR) analysis, relevant in the context of potential class imbalance, showed RF leading on the training set (PR-AUC = 0.9418), followed by LightGBM (0.9081; Figure 3C). On the internal validation set, LightGBM achieved the highest PR-AUC (0.9119), exceeding SVM (0.8114), RF (0.8300), LR (0.8229), and XGBoost (0.8673), highlighting its robustness across varying recall thresholds (Figure 3D).

Calibration, assessed via Brier score (lower is better), was strongest for LightGBM (0.0796) on the internal validation set, compared to XGBoost (0.0976) and RF (0.1043). RF showed the best calibration on the training set (0.0619), though LightGBM maintained well-calibrated predictions overall (Figures 3E, F).

**Figure 4:**
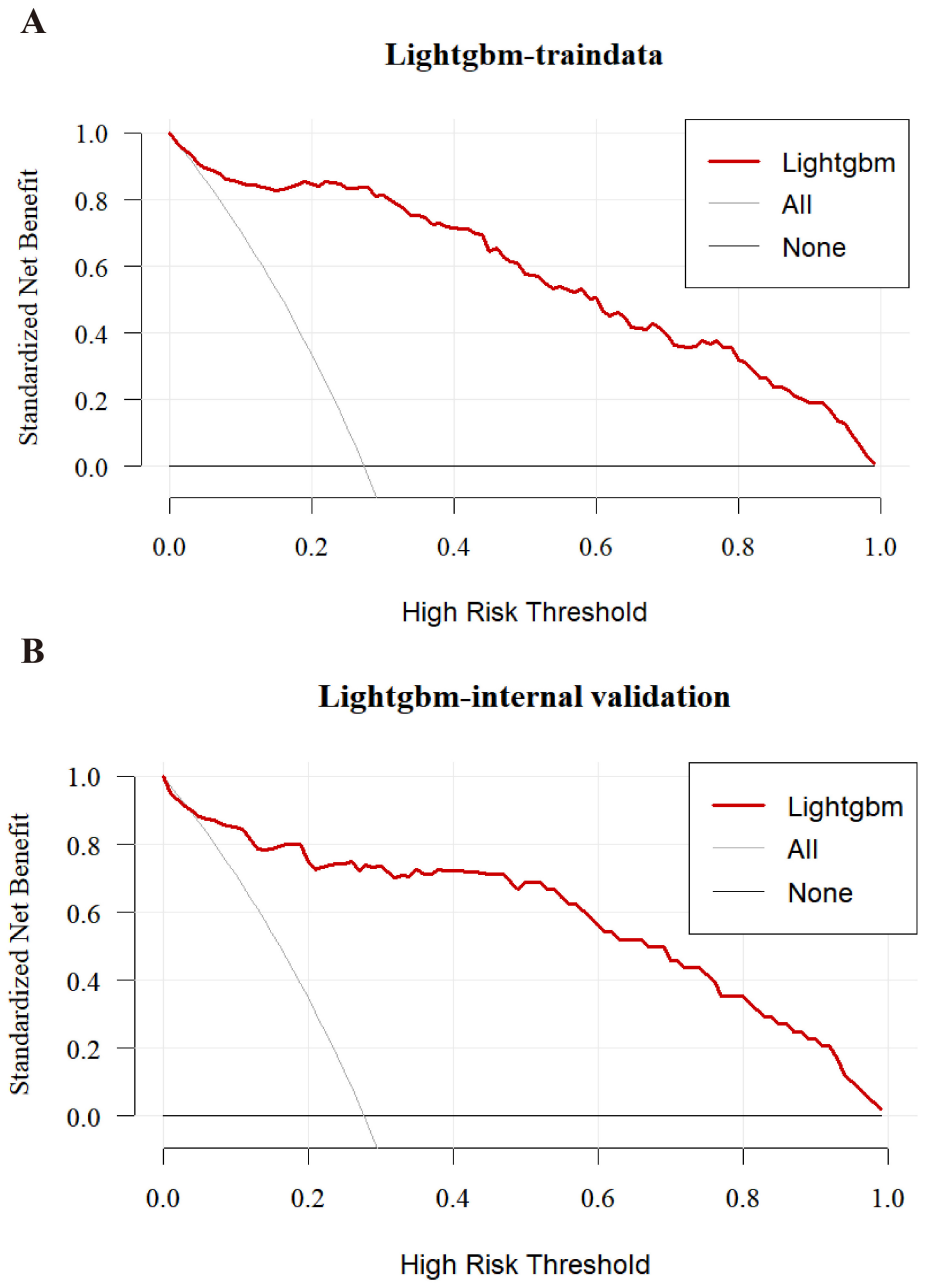
Decision curve analysis (DCA) for the LightGBM model. **(A)** DCA in train set; **(B)** DCA in internal validation set.

As summarized in Table 3, based on comprehensive evaluation on the internal test set, LightGBM exhibited the most favorable overall performance and was selected as the final model. Decision curve analysis (DCA) further confirmed the clinical utility of LightGBM, showing a consistently higher net benefit than “treat-all” or “treat-none” strategies across a wide range of threshold probabilities in both training and internal validation sets (Figures 4A, B).

**Table 3:** Comparative analysis of the performance outcomes across various machine learning models in internal validation cohort.

**Model**	**Accuracy**	**ROC-AUC**	**ROC-AUC 95% CI**	**Sensitivity**	**Specificity**
RF	0.850	0.891	0.830–0.952	0.813	0.864
Lightgbm	0.873	0.938	0.889–0.987	0.854	0.880
Xgboost	0.879	0.913	0.857–0.969	0.854	0.888
LR	0.844	0.882	0.816–0.947	0.792	0.864
SVM	0.809	0.883	0.818–0.948	0.833	0.800

### External validation of the LightGBM model

The generalizability and robustness of the LightGBM model were further evaluated using two independent external validation cohorts from different institutions.

**Figure 5:**
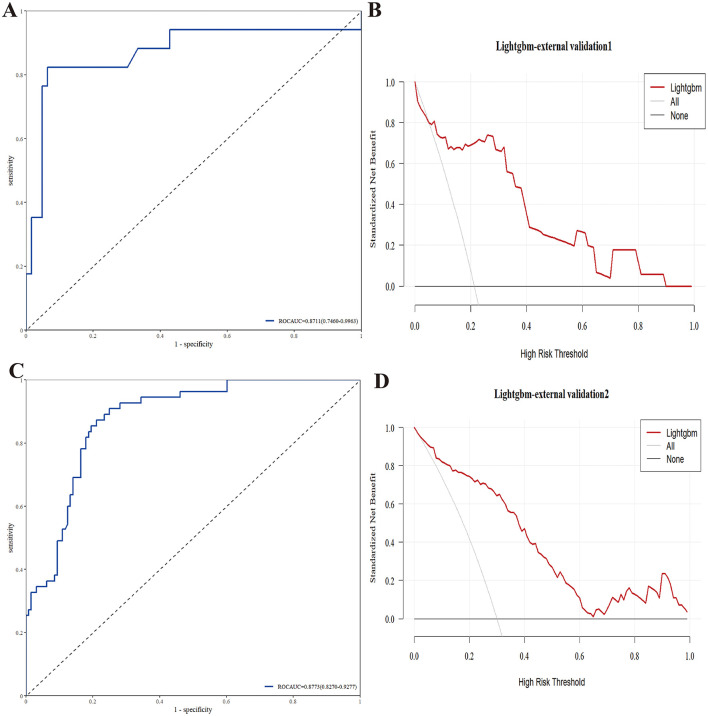
ROC curves and DCA for LightGBM model in two external validation cohorts. **(A, B)** ROC curves and DCA in external validation corhot 1; **(C, D)** ROC curves and DCA in external validation corhot 2.

In the external cohort 1 (*n* = 80), the model demonstrated strong discriminative ability, achieving an ROC AUC of 0.8711 (95% CI: 0.7460–0.9963; Figure 5A). DCA further confirmed the model's clinical utility, showing a higher net benefit across a wide range of threshold probabilities compared to the “treat-all” and “treat-none” strategies (Figure 5B).

Similar performance was observed in the external cohort 2 (*n* = 183), where the model attained an ROC AUC of 0.8773 (95% CI: 0.8270–0.9277; Figure 5C). The DCA results again indicated superior net benefit of the model over the entire clinically relevant threshold range, reinforcing its potential to support clinical decision-making (Figure 5D). The detailed performance results on the external validation set are provided in Supplementary Table S4.

These consistent results across two independent external datasets validate the strong performance and general applicability of the LightGBM model in diverse clinical settings, emphasizing its reliability and potential for real-world use.

### Model explanation

**Figure 6:**
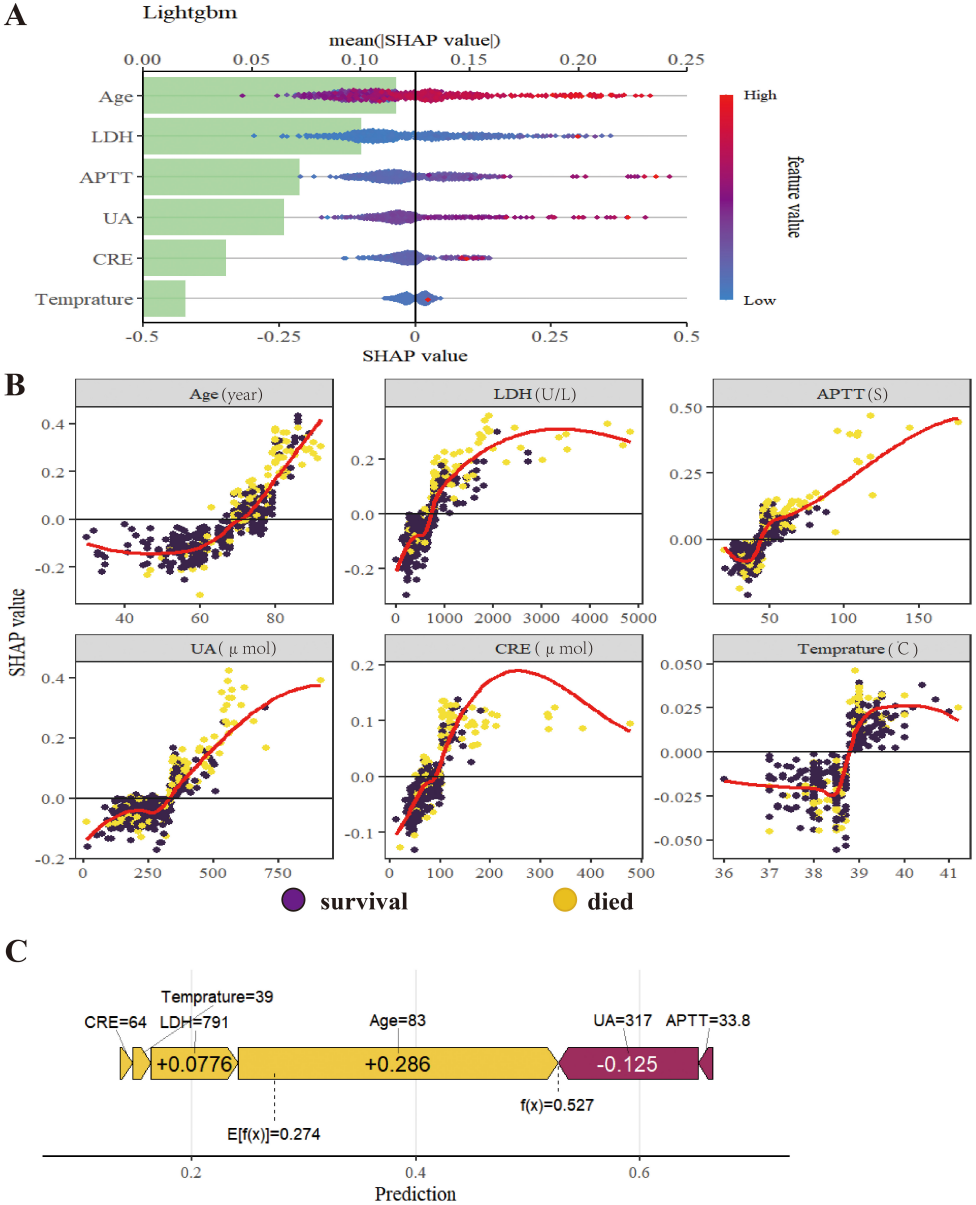
Model explanation by the SHAP method. **(A)** Global explanation of SHAP summary bar plot. (The mean SHAP values for feature importance) and summary dot plot (The impact of each feature on model output); **(B)** SHAP dependence plot. (Each dependence plot shows how a single feature affects the output of the prediction model, and each dot represents a single patient); **(C)** Local model explanation by the SHAP method. [Each arrow shows how a feature value (e.g., Age = 83) shifts the prediction from the base value to the final model output (f(x)). Yellow arrows indicate features that increase the prediction, while purple arrows indicate those that decrease it].

SHapley Additive exPlanations (SHAP) were used to interpret the LightGBM model. Globally, the summary plot (Figures 6A, B) indicated that Age was the most influential predictor, followed by LDH, APTT, UA, CRE, and Temperature. Age, LDH, CRE, UA, and APTT showed positive associations with mortality risk, while Temperature exhibited a more complex, non-linear relationship.

A local force plot (Figure 6C) illustrated the prediction for a representative high-risk patient (baseline risk = 0.274). For this specific patient, the feature Age (83 years) provided the strongest positive push (+0.286) toward a higher mortality risk score. The features LDH (791U/L), CRE (64 μmol) and a high temperature (39 °C), both with positive SHAP values, further contributed to elevating the risk. These risk-increasing effects were partially offset by a protective effect from UA = 317 μmol and APTT = 33.8s (−0.125). The sum of these contributions resulted in the final model prediction of 0.527 for this patient.

Collectively, these interpretability analyses confirm that the LightGBM model's decisions are driven by clinically relevant features.

### Implementation of web calculator

**Figure 7:**
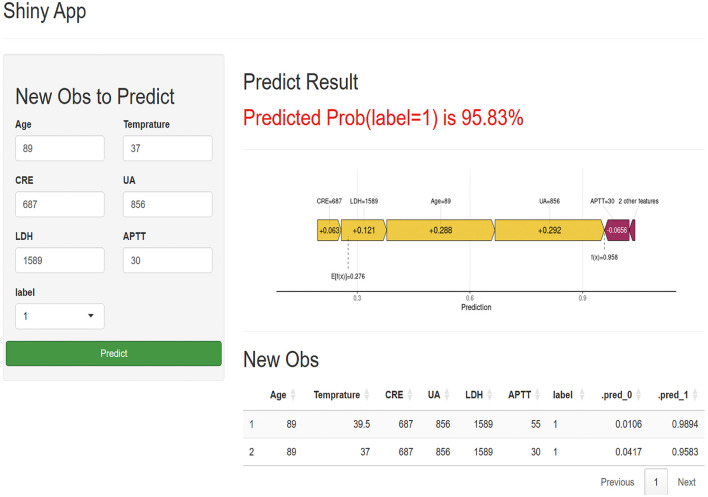
A web-based calculator APP for predicting prognosis in patients with SFTS. (Label = 1 means mortality risk).

To facilitate clinical translation, we developed a user-friendly web calculator using Shiny. The tool enables real-time individualized risk assessment based on the six feature inputs. It also provides an intuitive waterfall plot visualizing how each feature influences the prediction—using yellow to indicate factors increasing risk and purple for those decreasing it (Figure 7). In the APP, label = 1 indicates the risk of death, while label = 0 represents the probability of survival. The calculator is publicly available at: https://ml-model-1.shinyapps.io/online-model/.

## Discussion

This study developed and validated a LightGBM-based machine learning model for early prognosis prediction of SFTS, utilizing data from 834 patients across three medical centers in Anhui Province, China. Integrating six readily available clinical parameters at admission—Age, LDH, APTT, UA, CRE, and body temperature—the model demonstrated consistently strong discriminative performance across the training set (AUC = 0.960), internal validation set (AUC = 0.938), and two independent external validation sets (AUC = 0.871 and 0.877). By incorporating SHAP for model interpretability and deploying an online calculator, we established an integrated decision-support tool that combines high accuracy, transparency, and clinical utility, potentially addressing the dual challenges of delayed referral in primary care and early risk stratification in hospitals for SFTS management.

While the model maintained high predictive accuracy externally (AUC > 0.87), a modest attenuation compared to the internal validation was observed. This phenomenon is likely attributable to “spectrum bias” arising from the heterogeneity of patient populations and laboratory standards across institutions. Analysis of the external datasets (Supplementary Tables S1, S2) highlights these variations. Firstly, the mortality rate in External Cohort 1 was 18.8% (15/80), lower than the ~25% observed in the derivation cohort, indicating differences in disease prevalence. Secondly, the distribution of key predictors varied notably; for instance, while LDH levels showed a wide discriminatory gap in External Cohort 2 (median: 595 vs. 1,104 U/L for survivors vs. non-survivors), this margin was significantly narrower in External Cohort 1 (694 vs. 714 U/L). Such inconsistencies in feature distribution and case-mix likely increased the difficulty of classification. Nevertheless, the sustained performance across diverse settings confirms that the model relies on robust, pathophysiologically relevant predictors rather than site-specific noise.

The most notable strength of this study lies in its successful balance between model performance and clinical utility. Compared with previous models incorporating dozens of variables, our approach achieved comparable predictive accuracy using only six readily available clinical features. This approach is particularly valuable in primary care settings, where complex laboratory tests such as viral nucleic acid testing or cytokine profiling are often unavailable (15). Methodologically, our study represents a clear advance over existing approaches. In terms of feature parsimony, it demonstrates advantages over the XGBoost model by Xu et al. (seven features) and the UNION-SFTS model by Liu et al. (six features including viral load) (15, 16). Although viral load serves as a robust prognostic biomarker, its measurement is generally inaccessible in primary care institutions. By innovatively constructing a prediction model independent of such complex parameters, we ensure broad applicability in resource-limited settings. This rationale aligns with the research concept of He et al. (14) but is strengthened by our larger sample size (834 vs. 483 cases) and rigorous multicenter external validation, thereby providing higher-level evidence.

Model interpretability remains a critical bottleneck in clinical translation. The primary obstacle to the clinical adoption of machine learning models lies in their inherent “black-box” nature. This study successfully demystified the model's decision-making process through systematic SHAP analysis. Global explanations (via SHAP summary plots) enable clinicians to intuitively identify key predictors and their general impact on risk, while local explanations (achieved through force and waterfall plots) generate individualized “risk assessment reports” for specific patients. Our SHAP analysis not only quantified the contribution of each feature to the predictive outcomes but also elucidated their directional effects and interaction patterns.

The six predictors identified in this study collectively delineate the core pathophysiological landscape of severe SFTS. Theoretically, the clinical course of SFTS evolves through three distinct stages: the fever stage, the multi-organ dysfunction (MODS) stage, and the convalescent stage. Linking our predictors to these stages offers valuable insight into the dynamic evolution of prognosis. Although derived from admission data, the selected parameters serve as critical indicators of a patient's transition into the high-risk MODS stage.

Age, identified as the most significant predictor by SHAP, its impact is consistent with the majority of existing literature (3). The increased mortality in elderly patients is primarily driven by “immunosenescence”. Specifically, the age-related involution of the thymus leads to a reduced output of naïve T cells, impairing the host's ability to mount a rapid, specific immune response to the novel bunyavirus (22, 23). Furthermore, elderly individuals often exhibit “inflammaging”—a state of chronic, low-grade systemic inflammation. Upon infection, this predisposes them to a dysregulated cytokine storm and subsequent multi-organ failure, whereas younger patients with robust adaptive immunity can more effectively clear the virus. Other parameters further reflect this stage-dependent progression. LDH acts as a surrogate marker for the transition from viral replication to systemic inflammation; markedly elevated levels reflect widespread cell lysis and the intensity of the cytokine storm (15, 24, 25). APTT prolongation signals the onset of coagulopathy and potential disseminated intravascular coagulation (DIC), a hallmark of the late/severe stage (26, 27). CRE and UA: the simultaneous selection of both markers underscores the significant impact of SFTS on renal function. CRE levels directly reflect impaired glomerular filtration, while abnormal uric acid levels may stem from either reduced renal excretion or massive cell lysis due to high viral replication (analogous to the pathophysiology of tumor lysis syndrome) (28, 29). Their combination provides complementary information on renal injury and systemic metabolic disturbances. While the body temperature, it contrary to the conventional linear perception that “higher fever indicates greater severity,” SHAP dependence plots revealed a complex, non-linear relationship. The study found that very high body temperature (>39 °C) was associated with high risk, whereas some patients with poor outcomes presented with normal or even low body temperature. This phenomenon may indicate failure of thermoregulatory centers or the onset of severe sepsis, representing a danger signal of the terminal stage of the disease (30, 31). By capturing these stage-specific markers at admission, our model effectively identifies patients precipitating into the MODS stage, enabling preemptive intervention. This interpretability framework, which directly interfaces with clinical reasoning, establishes the foundation for physician trust in AI tools and represents a critical step in translating models from research publications to clinical bedside application.

Finally, the web-based online calculator developed in this study serves as the pivotal vehicle for translating our research into clinical value. Designed with a “ready-to-use” principle, it features an intuitive interface requiring minimal training. Its real-world application encompasses two primary scenarios: 1) In primary care settings (e.g., community health centers), physicians can input the six core parameters after obtaining basic laboratory test results for a suspected SFTS case. A high-risk prediction (e.g., >80%) provides an objective, quantifiable indicator for urgent referral, effectively mitigating delays caused by limited experience and streamlining the tiered healthcare pathway; 2) In regional medical centers or ICUs, the tool enables rapid risk stratification upon patient admission. A high-risk output triggers proactive clinical actions, including immediate ICU bed allocation, preemptive preparation of blood products (e.g., plasma, platelets), early assessment for continuous renal replacement therapy (CRRT), and timely proactive communication with families regarding critical illness. This facilitates a paradigm shift from reactive management to preemptive warning in SFTS patient care.

This study has several limitations: 1) the retrospective design may still involve residual confounding despite adjustments; 2) the model's generalizability is limited due to regional data from Anhui, China, requiring validation in other areas; 3) the static model, based on admission data only, cannot reflect dynamic clinical changes; 4) for handling missing data, we employed median/mode imputation. While this is a standard approach, advanced methods such as Multiple Imputation by Chained Equations (MICE) or k-Nearest Neighbors (k-NN) could potentially capture complex relationships between variables better. Given that we excluded features with >15% missingness and most variables have a missing rate of less than 2%, the impact is likely minimized, but future studies should consider these advanced techniques. 5) the online calculator's clinical effectiveness needs confirmation through prospective trials.

## Conclusion

This study has successfully developed and rigorously validated a prognostic prediction model for SFTS that utilizes only six routine clinical parameters obtainable at admission. Integrating exceptional predictive accuracy, profound interpretability, and practical clinical utility through an accessible online calculator, the model effectively supports decision-making across diverse healthcare settings. It provides objective referral guidance for primary care institutions while enabling early identification and proactive intervention for high-risk patients in tertiary hospitals. This tool offers a viable solution for establishing a tiered diagnosis and treatment system for SFTS, ultimately holding significant potential for improving patient outcomes.

## Data Availability

The original contributions presented in the study are included in the article/Supplementary material, further inquiries can be directed to the corresponding author.
