# Supplementary material for: Early mortality risk prediction in severe fever with thrombocytopenia syndrome using an interpretable machine learning model based on routine clinical parameters
Source: Front Public Health. 2026 Mar 9;14:1776344. doi: 10.3389/fpubh.2026.1776344 (PMC13006680; doi:10.3389/fpubh.2026.1776344)
Supplement: Supplementary file 1 [file Table_1.docx]

Table S1 **Summary of clinical variables and extent of missing data in the study cohort (N = 571).**

| **Variable** | **Missing Count** | **Missing Percentage (%)** |
| --- | --- | --- |
| Age | 2 | 0.35 |
| Temprature | 9 | 1.58 |
| PulseRate | 12 | 2.10 |
| SBP | 11 | 1.93 |
| DBP | 12 | 2.10 |
| WBC | 2 | 0.35 |
| NEUT# | 2 | 0.35 |
| LY# | 2 | 0.35 |
| MO | 2 | 0.35 |
| RBC | 2 | 0.35 |
| HGB | 2 | 0.35 |
| HCT | 2 | 0.35 |
| MCV | 2 | 0.35 |
| MCH | 2 | 0.35 |
| MCHC | 2 | 0.35 |
| PLT | 2 | 0.35 |
| CRP | 12 | 2.10 |
| TP | 1 | 0.18 |
| ALB | 1 | 0.18 |
| GLO | 1 | 0.18 |
| A/G | 1 | 0.18 |
| TBIL | 1 | 0.18 |
| ALT | 1 | 0.18 |
| AST | 1 | 0.18 |
| ALP | 1 | 0.18 |
| GGT | 1 | 0.18 |
| UREA | 3 | 0.53 |
| CRE | 3 | 0.53 |
| UA | 3 | 0.53 |
| GLU | 30 | 5.25 |
| LDH | 8 | 1.40 |
| CK | 27 | 4.73 |
| CK-MB | 27 | 4.73 |
| K | 9 | 1.58 |
| Na | 9 | 1.58 |
| CL | 9 | 1.58 |
| HCO3 | 9 | 1.58 |
| LPS | 23 | 4.03 |
| AMY | 23 | 4.03 |
| PT | 11 | 1.93 |
| PT | 11 | 1.93 |
| PT-INR | 11 | 1.93 |
| APTT | 11 | 1.93 |
| FIB | 11 | 1.93 |
| TT | 11 | 1.93 |
| DD | 11 | 1.93 |
| FDP | 11 | 1.93 |
| PCT | 2 | 0.35 |
| NRAP | 2 | 0.35 |
| AST/ALT | 1 | 0.18 |
| UCR | 4 | 0.70 |
| Sex | 0 | 0 |
| Hypertension | 0 | 0 |
| CHD | 0 | 0 |
| Diabetes | 0 | 0 |
