# Supplementary material for: Early mortality risk prediction in severe fever with thrombocytopenia syndrome using an interpretable machine learning model based on routine clinical parameters
Source: Front Public Health. 2026 Mar 9;14:1776344. doi: 10.3389/fpubh.2026.1776344 (PMC13006680; doi:10.3389/fpubh.2026.1776344)
Supplement: Supplementary file 2 [file Table_2.docx]

Table s2 Comparison of demographic and clinical characteristics between the Survived and died groups in external validation set 1.

| Feature | Survived(n=65) | Died(n=15) | P value |
| --- | --- | --- | --- |
| Age(year),(mean ± sd) | 66.723 ± 11.178 | 76.6 ± 9.3105 | 0.002 |
| Sex,n(%) |  |  | 0.407 |
| Femal | 38 (58.5%) | 7 (46.7%) |  |
| Man | 27 (41.5%) | 8 (53.3%) |  |
| Hypertension,n(%) |  |  | 0.177 |
| No | 40 (61.5%) | 12 (80%) |  |
| Yes | 25 (38.5%) | 3 (20%) |  |
| CHD,n(%) |  |  | 1.000 |
| No | 64 (98.5%) | 15 (100%) |  |
| Yes | 1 (1.5%) | 0 (0%) |  |
| Diabetes,n(%) |  |  | 0.396 |
| No | 52 (80%) | 14 (93.3%) |  |
| Yes | 13 (20%) | 1 (6.7%) |  |
| Temprature(℃)，median (IQR) | 39 (38.5, 39) | 38.5 (38.45, 38.9) | 0.048 |
| BPM, median (IQR) | 84 (74, 89) | 87 (79, 90) | 0.339 |
| SBP, mean ± sd | 119.28 ± 13.474 | 118.6 ± 12.252 | 0.859 |
| DBP, mean ± sd | 74.924 ± 9.2252 | 75.733 ± 8.7052 | 0.758 |
| WBC(10^9/L), median (IQR) | 2.55 (1.46, 3.93) | 2.06 (1.645, 2.46) | 0.187 |
| ANC(10^9/L), median (IQR) | 1.5 (0.98, 3.21) | 1.49 (1.235, 1.805) | 0.753 |
| ALC(10^9/L), median (IQR) | 0.66 (0.46, 1.05) | 0.34 (0.305, 0.5) | 0.003 |
| AMC(10^9/L), median (IQR) | 0.1 (0.05, 0.25) | 0.06 (0.035, 0.185) | 0.180 |
| RBC(10^12/L), median (IQR) | 4.28 (3.83, 4.66) | 3.75 (3.72, 3.89) | 0.010 |
| HGB(g/L), mean ± sd | 129.66 ± 17.149 | 123.53 ± 11.205 | 0.098 |
| HCT(%), median (IQR) | 0.402 (0.335, 0.445) | 0.355 (0.35, 0.3645) | 0.339 |
| MCV(fl), median (IQR) | 90.7 (86.4, 92.7) | 94 (91.15, 95.7) | 0.004 |
| MCH(pg), median (IQR) | 30.5 (29.7, 31.8) | 32.4 (31.55, 32.95) | 0.002 |
| MCHC(g/L), mean ± sd | 340.15 ± 12.044 | 342.6 ± 16.66 | 0.513 |
| PLT(10^9/L), median (IQR) | 57 (42, 72) | 64 (45, 70) | 0.448 |
| CRP, median (IQR) | 4.59 (1.09, 7.89) | 3.84 (1.93, 7.42) | 0.975 |
| TP(g/L), mean ± sd | 62.797 ± 6.2318 | 64.913 ± 9.309 | 0.287 |
| ALB(g/L), median (IQR) | 38.1 (34.6, 41.2) | 39.7 (33.55, 42.45) | 0.554 |
| GLO(g/L), median (IQR) | 25.5 (20.7, 29.8) | 27.1 (23.8, 31.15) | 0.312 |
| A/G, median (IQR) | 1.51 (1.29, 1.87) | 1.35 (1.26, 1.74) | 0.448 |
| TBIL(μmol/L), median (IQR) | 9.9 (7.8, 12.4) | 10.1 (9.4, 13.05) | 0.490 |
| ALT(U/L), median (IQR) | 44 (30, 92) | 33 (20, 44.5) | 0.046 |
| AST(U/L), median (IQR) | 95 (70.5, 178) | 85 (37, 124) | 0.225 |
| ALP(U/L), median (IQR) | 60.8 (48, 70.2) | 57 (51.5, 73) | 0.810 |
| GGT(U/L), median (IQR) | 23 (15, 56) | 19 (14, 26) | 0.077 |
| UREA(mmol/L), median (IQR) | 6.4 (5.2, 8.1) | 7.22 (6.55, 9.2) | 0.029 |
| CRE(μmol/L), median (IQR) | 78 (65, 97) | 93 (76.95, 109.95) | 0.027 |
| UA(μmol/L), median (IQR) | 237 (187, 261) | 249.4 (195, 270.5) | 0.032 |
| GLU(mmol/L), median (IQR) | 7.2 (6.46, 9) | 6.95 (6.155, 14.1) | 0.693 |
| LDH(U/L), median (IQR) | 694.21 (487, 714.21) | 714.21 (577, 734.21) | 0.013 |
| K(mmol/L), mean ± sd | 3.644 ± 0.4762 | 3.7767 ± 0.59513 | 0.357 |
| Na(mmol/L), mean ± sd | 133.35 ± 4.0756 | 132.05 ± 4.082 | 0.268 |
| CL(mmol/L), median (IQR) | 100 (99, 102.93) | 98 (97.195, 101) | 0.091 |
| HCO3(mmol/L), median (IQR) | 24 (22, 26) | 23 (22.5, 26.35) | 0.887 |
| LPS(U/L), median (IQR) | 114 (52, 162.23) | 105 (76, 162.23) | 0.955 |
| AMY(U/L), median (IQR) | 102 (65, 118.4) | 101 (82, 117.2) | 0.723 |
| PT(s), mean ± sd | 12.248 ± 1.2712 | 11.933 ± 0.9676 | 0.371 |
| PT%, mean ± sd | 92.67 ± 15.783 | 95.227 ± 13.414 | 0.564 |
| PT-INR, mean ± sd | 1.043 ± 0.11593 | 1.016 ± 0.087652 | 0.399 |
| APTT(s), median (IQR) | 34.7 (33.44, 39.3) | 38.2 (35.57, 43.44) | 0.032 |
| FIB(g/L), median (IQR) | 2.95 (2.48, 3.39) | 2.61 (2.205, 3.835) | 0.475 |
| TT(s), median (IQR) | 19.9 (17.8, 22.6) | 20 (17.05, 24.375) | 0.961 |
| DD(μg/ml), median (IQR) | 2.62 (1.3, 4.31) | 3.42 (2.11, 10.52) | 0.160 |
| PCT(ng/ml), median (IQR) | 0.32 (0.13, 0.67224) | 0.36 (0.226, 0.67224) | 0.615 |
| NRAP, median (IQR) | 1.0189 (0.63865, 1.8326) | 1.9471 (1.8019, 2.067) | < 0.001 |
| AST/ALT, median (IQR) | 2.25 (1.7143, 2.7143) | 2.2857 (1.85, 3.0592) | 0.381 |
| UCR, mean ± sd | 20.658 ± 4.9452 | 22.887 ± 4.9882 | 0.120 |

**Abbreviations: IQR: Interquartile Range；sd: standard deviation; CHD: Coronary Heart Disease; BPM: Beats Per Minute; SBP: Systolic Blood Pressure; DBP: Diastolic Blood Pressure; ANC: Absolute Neutrophil Count; ALC: Absolute Lymphocyte Count; AMC: Absolute Monocyte Count; A/G: Albumin to Globulin Ratio; UCR:** (**UREA to CRE)*250**
