# Supplementary material for: Early mortality risk prediction in severe fever with thrombocytopenia syndrome using an interpretable machine learning model based on routine clinical parameters
Source: Front Public Health. 2026 Mar 9;14:1776344. doi: 10.3389/fpubh.2026.1776344 (PMC13006680; doi:10.3389/fpubh.2026.1776344)
Supplement: Supplementary file 3 [file Table_3.docx]

Table s3 Comparison of demographic and clinical characteristics between the Survived and died groups in external validation set 2.

| Feature | Survived(n=134) | Died(n=49) | P value |
| --- | --- | --- | --- |
| Age(year),(mean ± sd) | 66 (56.25, 72.75) | 76 (69, 79) | < 0.001 |
| Sex,n(%) |  |  | 0.057 |
| Femal | 76 (56.7%) | 20 (40.8%) |  |
| Man | 58 (43.3%) | 29 (59.2%) |  |
| Hypertension,n(%) |  |  | 0.096 |
| No | 99 (73.9%) | 30 (61.2%) |  |
| Yes | 35 (26.1%) | 19 (38.8%) |  |
| CHD,n(%) |  |  | 1.000 |
| No | 133 (99.3%) | 48 (98%) |  |
| Yes | 1 (0.7%) | 1 (2%) |  |
| Diabetes,n(%) |  |  | 0.552 |
| No | 119 (88.8%) | 45 (91.8%) |  |
| Yes | 15 (11.2%) | 4 (8.2%) |  |
| Temprature(℃)，median (IQR) | 37.5 (37.1, 38.5) | 39 (38.3, 39.1) | 0.015 |
| BPM, median (IQR) | 80 (70, 90) | 84 (76, 98) | 0.080 |
| SBP, mean ± sd | 110.38 ± 17.378 | 119.3 ± 18.63 | 0.003 |
| DBP, mean ± sd | 69 (63, 75.75) | 70.973 (67, 79) | 0.078 |
| WBC(10^9/L), median (IQR) | 2 (1.4075, 3.63) | 2.75 (2, 5.27) | 0.071 |
| ANC(10^9/L), median (IQR) | 1.38 (0.7725, 2.3375) | 2.09 (1.39, 3.16) | 0.008 |
| ALC(10^9/L), median (IQR) | 0.58 (0.4, 0.8925) | 0.5 (0.32, 0.9) | 0.510 |
| AMC(10^9/L), median (IQR) | 0.11 (0.06, 0.2375) | 0.07 (0.05, 0.24) | 0.248 |
| RBC(10^12/L), median (IQR) | 4.205 (3.82, 4.6325) | 4.35 (3.98, 4.76) | 0.097 |
| HGB(g/L), mean ± sd | 127 (115.5, 138) | 134 (124, 148) | 0.010 |
| HCT(%), median (IQR) | 38.1 (35.125, 40.875) | 40.2 (37.2, 43.6) | 0.011 |
| MCV(fl), median (IQR) | 90.3 (87.625, 93.5) | 92.5 (89.5, 94.2) | 0.014 |
| MCH(pg), median (IQR) | 30.36 ± 1.6383 | 30.906 ± 1.5212 | 0.043 |
| MCHC(g/L), mean ± sd | 336.5 (328, 344) | 336 (326, 342) | 0.466 |
| PLT(10^9/L), median (IQR) | 53.5 (37.25, 67.5) | 38 (27, 51) | < 0.001 |
| CRP, median (IQR) | 4.635 (1.8075, 8.3802) | 7.93 (4.07, 15.5) | < 0.001 |
| TP(g/L), mean ± sd | 60.6 (56.65, 65.575) | 58.9 (56.7, 63.9) | 0.313 |
| ALB(g/L), median (IQR) | 35.281 ± 4.6059 | 32.741 ± 4.8917 | 0.001 |
| GLO(g/L), median (IQR) | 25.65 (22.025, 28.6) | 26.9 (23.8, 30.1) | 0.039 |
| A/G, median (IQR) | 1.4187 ± 0.33652 | 1.2433 ± 0.31327 | 0.002 |
| TBIL(μmol/L), median (IQR) | 8.85 (7.2, 11.95) | 10.4 (8.4, 13.4) | 0.028 |
| ALT(U/L), median (IQR) | 58.5 (33.25, 106) | 82 (56, 179) | < 0.001 |
| AST(U/L), median (IQR) | 127.65 (67.8, 284.32) | 314.2 (143, 724) | < 0.001 |
| ALP(U/L), median (IQR) | 62.8 (51.25, 80) | 73.2 (57, 97) | 0.031 |
| GGT(U/L), median (IQR) | 28 (19, 54) | 51 (29, 96.4) | 0.002 |
| UREA(mmol/L), median (IQR) | 6 (4.425, 7.4) | 8.3 (6.4, 14.3) | < 0.001 |
| CRE(μmol/L), median (IQR) | 69 (60.625, 81) | 98 (73.1, 145.9) | < 0.001 |
| UA(μmol/L), median (IQR) | 237.45 (170.25, 296.75) | 300.6 (193.2, 401.5) | 0.007 |
| GLU(mmol/L), median (IQR) | 6.995 (5.915, 9.3525) | 7.42 (6.14, 10.58) | 0.164 |
| LDH(U/L), median (IQR) | 595 (380.85, 1104.1) | 1104.1 (464, 1974) | 0.002 |
| K(mmol/L), mean ± sd | 3.625 (3.2325, 3.9875) | 4.01 (3.51, 4.46) | < 0.001 |
| Na(mmol/L), mean ± sd | 132.13 ± 4.651 | 133.48 ± 5.5571 | 0.103 |
| CL(mmol/L), median (IQR) | 99.18 (97, 102.53) | 100.8 (97.83, 104.4) | 0.097 |
| HCO3(mmol/L), median (IQR) | 23.254 ± 3.4522 | 20.19 ± 4.9357 | < 0.001 |
| LPS(U/L), median (IQR) | 391.99 (150.06, 391.99) | 391.99 (331.59, 391.99) | 0.082 |
| AMY(U/L), median (IQR) | 145.97 (99.75, 145.97) | 145.97 (145.97, 145.97) | 0.027 |
| PT(s), mean ± sd | 11.95 (11.3, 13.2) | 12.6 (11.4, 13.5) | 0.060 |
| PT%, mean ± sd | 103.99 (94.31, 122.78) | 100.11 (84.46, 109.91) | 0.042 |
| PT-INR, mean ± sd | 0.97 (0.9, 1.03) | 1.02 (0.93, 1.09) | 0.016 |
| APTT(s), median (IQR) | 44.2 (38.825, 49.95) | 53.6 (43, 64.7) | < 0.001 |
| FIB(g/L), median (IQR) | 2.2798 (1.98, 2.575) | 2.03 (1.81, 2.35) | 0.024 |
| TT(s), median (IQR) | 20.1 (18.3, 24.775) | 26 (20.8, 40.1) | < 0.001 |
| DD(μg/ml), median (IQR) | 1.97 (1.0675, 4.5075) | 3.89 (2.28, 6.58) | < 0.001 |
| PCT(ng/ml), median (IQR) | 0.17 (0.1, 0.52052) | 0.52052 (0.25, 2.01) | < 0.001 |
| AST/ALT, median (IQR) | 2.34 (1.7925, 2.9325) | 2.9 (2.19, 4.33) | < 0.001 |
| UCR, mean ± sd | 20.957 ± 6.1351 | 22.266 ± 6.5969 | 0.212 |

**Abbreviations: IQR: Interquartile Range；sd: standard deviation; CHD: Coronary Heart Disease; BPM: Beats Per Minute; SBP: Systolic Blood Pressure; DBP: Diastolic Blood Pressure; ANC: Absolute Neutrophil Count; ALC: Absolute Lymphocyte Count; AMC: Absolute Monocyte Count; A/G: Albumin to Globulin Ratio; UCR:** (**UREA to CRE)*250**
