# Supplementary material for: Early mortality risk prediction in severe fever with thrombocytopenia syndrome using an interpretable machine learning model based on routine clinical parameters
Source: Front Public Health. 2026 Mar 9;14:1776344. doi: 10.3389/fpubh.2026.1776344 (PMC13006680; doi:10.3389/fpubh.2026.1776344)
Supplement: Supplementary file 4 [file Table_4.docx]

| **Cohort** | **Accuracy**  **(95% CI)** | **ROC-AUC**  **(95% CI)** | **Sensitivity**  **(95% CI)** | **Specificity**  **(95% CI)** | **PR-AUC**  **(95% CI)** |
| --- | --- | --- | --- | --- | --- |
| External corhort 1 | 0.9125  (0.85-0.975) | 0.8711  (0.7459-0.9963) | 0.8235  (0.6364-1.0) | 0.9365  (0.874-0.9847) | 0.733  (0.4861-0.9227) |
| External corhort 2 | 0.8032  (0.7486-0.8579) | 0.8773(0.827-0.9277) | 0.8909  (0.7943-0.9655) | 0.7656  (0.6944-0.8308) | 0.7509(0.6331-0.8418) |

Table s4. The detailed performance results of ligthGBM model on the external validation set
